# Effects of physiotherapy on balance and unilateral vestibular hypofunction in vertiginous elderly

**DOI:** 10.1186/1755-7682-7-8

**Published:** 2014-02-27

**Authors:** Paulo Roberto Rocha Júnior, Amanda da Silva Peres, Fernando Pereira Garbi, Ana CF Frizzo, Vitor E Valenti

**Affiliations:** 1 Faculdades Adamantinenses Integradas, Via de Acesso Francisco Bellusci, 1000, Adamantina, SP 17800-000, Brazil; 2 Departamento de Fonoaudiologia, Faculdade de Filosofia e Ciências, UNESP, Av. Hygino Muzzi Filho, 737, Marília, SP 17525-900, Brazil

**Keywords:** Aged, Postural balance, Physical therapy modalities, Vestibular diseases

## Abstract

**Background:**

We aimed to analyze the effect of a physical therapy protocol on unilateral vestibular hypofunction and overall balance in elderly with vertigo.

**Methods:**

The study included nine subjects, four male subjects (68.5 ± 11.09 years old) and five females (72.4 ± 7.09 years old). It was used the performance-oriented Mobility Assessment (POMA), to evaluate the balance and the Unterberger – Fukuda test for analysis of unilateral vestibular dysfunction through rotations and displacements of the body. We developed and applied a structured physical therapy protocol, consisting of group exercises.

**Results:**

It was observed that after the protocol, all participants improved balance (33.9 ± 5.1 vs. 47.3 ± 7.6, p < 0.0001) and displacement (111.1 ± 38.0 vs. 53.3 ± 34.6, p = 0.0001). However, it was not found significant differences for rotation.

**Conclusion:**

The proposed protocol has contributed to an improvement in balance and vestibular dysfunction of the aged.

## Background

Maintaining upright posture becomes a challenge with increasing age, whereas the instability is related, among other components, with the involvement of the vestibular apparatus. There is a reduction in labyrinthine hair cells receptor density and the amount of vestibular ganglion cell receptor related to aging [1]. The vestibular system is considered a benchmark for effectiveness of maintaining balance, compared with the visual and somatosensory system. When there is no integration of visual information, proprioceptive and labyrinthine, the balance will be affected [2].

Dizziness is one of the most common symptoms in the elderly and covers various sensations of balance disorder. Other common symptoms include dizziness, instability, imbalance and spatial disorientation, and these contribute potentially to the falls [3].

Due to the large number of elderly subjects with symptoms of dizziness and lack of therapeutic programs to this specific population, we evaluated the effect of a structured physiotherapy protocol on balance and peripheral vestibular dysfunction in elderly with vertigo.

## Methods

### Study population

The study was conducted in indoor sports court, twice a week, for about an hour over a period of four months in the town of Adamantina, SP, Brazil. The sample consisted of individuals aged over 60 years old of both sexes with a diagnosis of non-specific peripheral vestibular disease and/or symptoms of dizziness for more than three months. The study excluded patients with restrictive diseases that avoided the achievement of pre-established exercises with degenerative, neoplastic, individuals who used assistive devices for walking and/or who did not agree with the prerogative of the study. For the selection of the sample it was delivered to the medical clinics in the city of adamantina a letter of request for referral of individuals to the physiotherapy protocol. This program was called Vertigo!. All participants were instructed on the procedures and objectives of the study and signed an informed consent form. All procedures were approved by the ethical committee in research of our Institution.

### Unterberger-Fukuda test

**Figure 1:**
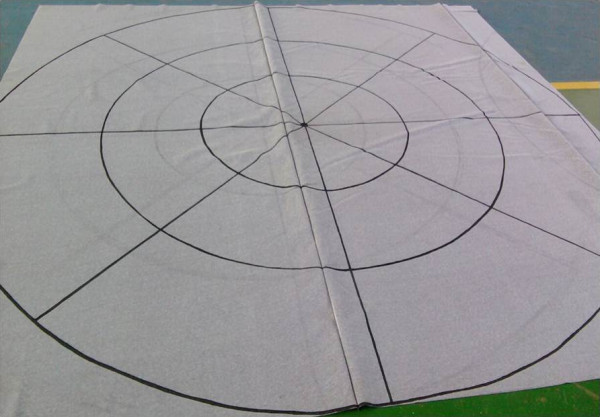
“Carpet” developed by researchers to evaluate the rotation and displacement of the patients.

The test was performed on three concentric circles drawn on a carpet whose radii are 0.5 m apart from each other. These circles were divided into 12 equal parts, by lines that cross the center, forming an angle of 30°. The patient moved, bringing their knees about 45° without moving, running 60 steps (one per second) from the center, with arms outstretched and closed eyes. It was considered pathological result when there was greater than 1 m displacement and/or rotation higher than 30°. This test is useful in monitoring patients with peripheral diseases during treatment because it provides signs of unilateral vestibular dysfunction [4]. In order to systematize and ensure greater reliability in the test of Unterberger-Fukuda we developed a “carpet” with the above measures (Figure 1).

### Performance-oriented mobility assessment (POMA)

It aims to detect risk factors for falls in the elderly. The test is divided in two parts: one evaluates the balance and the other evaluates gait. The maneuvers include eight equilibrium positions in destabilization of the gravity center. The marching maneuvers also included eight items made through sequential activities in a small path running with simple criterion score. The test result is given in score when higher the score achieved, the better the performance in the test. The maximum score is fifteen and thirteen to balance for gait [4].

### Procedure

A protocol was created for vestibular rehabilitation exercises based on studies of Cawthorne and coworkers [5]. These authors developed a sequence of exercises originally indicated for symptoms of dizziness caused by vestibular dysfunction. It is indicated to improve vestibular adaptation by stimulating the vestibular-ocular reflex and tolerance of head movements without triggering the symptoms of dizziness. It was prioritized exercises that addressed proprioception, coordination, and fundamentally the movement (Additional file 1). This protocol was submitted to two judges with expertise in the area of vestibular dysfunction in order to ensure its reliability. The criticisms and suggestions of each judge were analyzed and contributed to the final version of the protocol. The program lasted four months, totaling 30 sessions. The variables were measured before and one week after the physiotherapy protocol.

### Statistical analysis

Standard statistical methods were used for the calculation of means and standard deviations. Normal Gaussian distribution of the data was verified by the Shapiro-Wilk goodness-of-fit test (z value >1.0). Regarding the first protocol for parametric distributions, we applied the paired Student *T* test and for non-parametric distributions we used the paired Wilcoxon test. Differences were considered significant when the probability of a Type I error was less than 5% (p < 0.05). We used the Software GraphPad StatMate version 2.00 for Windows, GraphPad Software, San Diego California USA.

## Results

Table 1 shows that the physiotherapy protocol significantly improved balance (p < 0.05) and anterior displacement (p < 0.05), while there was no significant change in the rotation.

**Table 1:** Mean values, standard deviation and percentage of balance, anterior displacement and rotation before and after the physiotherapy intervention

**Variable**	**Before physiotherapy protocol**	**After physiotherapy protocol**	**p**
**Balance**	33.9 ± 5.1	47.3 ± 7.6	<0.0001
**Anterior displacement**	111.1 ± 38.0	53.3 ± 34.6	0.0001
**Rotation**	43.3 ± 21.1	31.1 ± 30.6	0.11

## Discussion

In this study we aimed to evaluate the effects of a physiotherapy protocol on balance, anterior displacement and rotation in elderly with vertigo. As a main finding, it improved balance and anterior displacement.

Neuroplasticity occurs when exercise sensory disturbances generated by the adjustment requests the need to be taken by the central nervous system [6]. Therefore, the development of an exercise protocol that addresses primarily the repetition, favors the phenomenon of habituation that through sensory stimulation promotes vestibular compensation due to neuronal plasticity neuronal. This phenomenon is obtained by performing repetitive movements, which attenuates the vestibular response and the amplitude of nystagmus. The repetition, in addition to promoting adaptation to movement, stimulates the peripheral sensory organ, responsible for creating new automatic body balance [1].

We reported benefic effects of this physiotherapy protocol on elderly with vertigo. The vestibular rehabilitation exercises, according to Ribeiro et al. [5], restores the balance through the stimulation and acceleration of compensatory natural means, which involves movements of the head, neck and eyes. In developing the protocol to the exercises it should present evolution feature, with respect to the level of difficulty of the exercises, and physical therapy, with respect to proprioception and movement.

Similarly, Steadman et al. [7], who had a group of seniors for balance training consisting of a series of repetitive tasks of increasing difficulty, found that the program significantly improved balance and mobility of the sample.

The proposed protocol by our group included a proprioception circuit. To Cozzani et al. [2], the elderly are less able to adapt to additional disturbance during ambulation, requiring, thus, a greater emphasis on gait training with obstacles. As performed at the beginning of the program, Miniti et al. [8] reported the importance of guidance and information on training exercises of the vestibular-ocular reflex and somatosensory system. Taken together, those references support our protocol proposed in this study

Overall, there were satisfactory results regarding improvement in unilateral asymmetry peripheral of the vestibular system and balance of the sample. In this context, it was [9] reported that physiotherapy prevention programs contribute to improving balance, reducing consequently falls, therefore, confirming the importance of these programs for the elderly.

Proposals interventional study and its results, after the development and application of exercise protocol could serve as a basis for therapeutic measures to be applied in other institutions, promoting improved balance and possible vestibular hypofunctions in the elderly population.

## Conclusion

The structured physiotherapy protocol for vestibular rehabilitation contributed to an improvement of balance and anterior displacement in elderly with vertigo.

## Competing interests

The authors declare that they have no competing interests.

## Authors’ contributions

PRRJ, ASP, FPG, ACFF and VEV participated in the acquisition of data and revision of the manuscript. PRRJ, ASP and FPG conceived the study, determined the design, interpreted the data and drafted the manuscript. CF PRRJ, ASP, FPG, ACFF and VEV interpreted the data and drafted the manuscript. All authors read and gave final approval for the version submitted for publication.

## Supplementary Material

Additional file 1 Description of the physiotherapy protocol developed by the group. Click here for file
