# Supplementary material for: Effects of physiotherapy on balance and unilateral vestibular hypofunction in vertiginous elderly
Source: Int Arch Med. 2014 Feb 27;7:8. doi: 10.1186/1755-7682-7-8 (PMC3938970; doi:10.1186/1755-7682-7-8)
Supplement: Additional file 1 — Description of the physiotherapy protocol developed by the group. [file 1755-7682-7-8-S1.docx]

**Additional file 1.** Description of the physiotherapy protocol developed by the group.

| **1ª Step** (Movements of eyes and head, first slowly, then faster). |
| --- |
| Patient standing on a mat, looking up and down. (50 reps) |
| Patient standing on a mat, look to the right and to the left. (50 reps) |
| Patient standing on a mat, zoom in and out the index finger of the face, staring at him. (50 reps) |
| Patient standing on a mat, moving the head (slowly and then faster) to the right and to the left with your eyes open, focus on a fixed point. (50 reps) |
| Patient standing on a mat, flex and extend your head (slowly and then faster) up and down with your eyes open, focusing on a fixed point. (50 reps) |
| **2ª Step** (Movement of head and body) |
| Patient places an object on the ground. Staring at it, he/she picks up the object and rises above the head, and then places the object on the ground again. ( 30 reps ) |
| Shrug and make circular movements with the shoulder , looking at a fixed point stuck to the wall . ( 30 reps ) |
| Leaning forward and pass an object back and front of the knees, always looking closely at the object . ( 30 reps ) |
| Patient standing on a mat performs opening and closing movements of the articulation of the mouth , with the patient looking closely at a point mobile manipulated by the therapist . ( 30 reps ) |
| Patient standing on a mat performs movements of lateral deviation of the jaw, looking to the horizon. ( 30 reps ) |
| Patient walks three steps forward performing massage in masticatory muscles associated with flexion and extension of the head, with eyes fixed on the therapist. ( 30 reps ) |
| Patient sitting with eyes open, hands resting on the knee flexed, trunk extended and lateral- flexion with the trunk, staring at a point on the wall. ( 30 reps ) |
| **3ª Step** (Standing and static exercises) |
| Sit and stand, sit and stand back up, with eyes fixed on a point on the ceiling. (30 reps) |
| Sit and stand, sit and stand back with arms crossed and eyes closed. (30 reps) |
| Patient standing performs a 360 ° turn to the right, returning to standing position (30 reps) |
| Patient standing performs a 360 ° turn to the left, at the standing position. (30 reps) |
| Throw a small ball from one hand to the other, above the horizon, fixing the eyes on the ball when it moves toward each hand. (50 reps) |
| Patient standing with a member of the front of the other, the patient plays a ball from one hand to the other, down the legs, alternately, fixing his/her gaze on the ball. (30 reps) |
| **4ª Step** (Dinamic and static exercises) |
| Patient standing on a mat, with the eyes open, hands behind trying to bring the shoulder, the patient holds trunk flexion and extension and lateral flexion of the trunk. (30 reps) |
| Therapist standing in front of the patient with his/her finger to the side and the head of the individual on the other side with eye-gaze of the patient in relation to the therapist's finger. (50 reps) |
| Patient standing on a mat throw the ball from one hand to the other, following the movement of the ball with the eyes. (50 reps) |
| Patient walks on a straight line drawn on the floor, staring at a fixed point on the wall. (50 reps) |
| Patient walk in a straight line looking up and down. (30 reps) |
| **5ª Step** |
| Patient walking throw the ball from one hand to the other, fixing the eyes on the ball. (30 reps) |
| Patient goes up and down on the steppe, open eyes, fixed on a target that is on the hand of the therapist. (50 reps) |
| Patient must balance on a single member, with eyes open and fixed on a point on the wall. (30 reps) |
| Patient walking on a straight line overlapping the feet facing each other, hands behind the trunk, focuses on a point in the distance. (30 reps) |
| Patient walks at a distance of three to four meters fixing their gaze on a fixed point and then will go back to steps coast to the starting point of the walk. (30 reps) |
| **6ª Step** |
| Patient sits up with the eyes closed and arms folded. (30 reps) |
| Patient standing with eyes closed, hands behind trying to bring the shoulder, the patient perform trunk flexion and extension and lateral flexion of the trunk (30 reps) |
| Patient standing on a mat is supported on a single limb with eyes closed (30 reps) |
| Walk on mat staring at a poster that is away, the patient should read the sentences written on the poster. (30 reps) |
| Patient sitting, grabs an object that is on the floor, with the eyes fixed on the therapist. (30 reps) |
| **7ª Step** |
| Patient is sitting up, and on a mat performs a turn of 360 ° and sit back (30 reps) |
| Patient sitting gets up and walks fixing his/her gaze on a straight line drawn on the ground and the end of the line the patient picks up an object on the ground and kicking with steps back to the starting point (30 reps) |
| Patient walking on a circuit with obstacles circuit performs the reading posters that is done on the path. (30 reps) |
| Patient performs circular movements with the hoop using the hands by setting the look on the hoop. (30 reps) |
| Patient facing the wall with a ball tries to hit the fixed point to be drawn on the wall, focusing always look at this point. (50 reps) |
| **8ª Step** |
| Patient to walk in a straight line hitting the ball on the ground fixing the gaze on a point that is ahead. (30 reps) |
| Patient standing throws a hula hoop on the air, and catches with the left hand and vice versa, always fixing the gaze on the movement of the bow. (30 reps) |
| Patient standing holding hula hoop flat on the floor, roll the hoop in the courtyard with the right hand and then with the left hand, keeping the eyes fixed on a distant point on the wall. (30 reps) |
| Standing on an exercise mat, holding the hoop in front of the body with both hands, pass inside the arc, hold hands and even keeping his gaze on the therapist. (30 reps) |
| **9ª Step** |
| Patient standing with hoop on the hand, rotate the hoop on the wrist, to the right and to the left with both hands. (50 reps) |
| Patient standing, legs apart, flexed at 90º on a mat. With the hoop above the head, secure the two hands. Flex the trunk to the left and right side, with arms outstretched and pasted on the head, and gaze on a point in the distance. (50 reps) |
| Patient standing, legs apart and flexed at 90º on a mat, with the hoop above your head safe by both hands, arms extended and glued on the head. Bend your arms until the arc reaches the shoulder height, while flexing his legs as if he were sitting with their eyes fixed on the therapist, return to starting position. (50 reps) |
| Walking on mats, staring at a poster that will be away, the patient should read the sentences written on the poster. (30 repetitions). |
| **10ª Step** |
| Patient lying on a mattress with a ball on the back of the neck. Keep your legs bent and draw a flower with each petal, first with the nose, then draw the center of the flower with the gaze on a point that is on the ceiling. (30 reps) |
| Patient lying on a mattress, with the ball on the back of the neck. Keep your legs bent. Performs denial movement with his head. (50 reps) |
| With two hula hoops on the ground with some distance between them, with the patient within each of them, throw a ball at each other, unable to leave their hoop to catch the ball. (30 reps) |
| **11ª Step** |
| Patients standing in pairs, on a mat, one of which holds a bat making smooth movements with changes of direction, and its accompanying pair moves slowly with eyes fixed on the bat. (30 reps) |
| Patients in pairs, on a mat, holding hands in front of the wall, the therapist with a laser or flashlight moves slowly points of light on the wall, the patient follows the movements only look and then monitors the movements look and head. (50 reps) |
| Patients in double, one with his back to the other with a ball stuck between them. Putting a song that may contain variations of pace (slow, moderate and fast) so that dance without letting the ball fall to the ground, maintaining a look on a fixed point on the wall. (30 reps) |
| **12ª Step** |
| Patient follow up walking about a'' eight'' which will be drawn on the ground, focusing intently straight segment. (50 reps) |
| Patient with the knees bended on a mat, focusing on a fixed point, performs movements circumduction shoulders. (50 reps) |
| Patient stands following a straight line drawn on the floor, walks marching, focusing on an object at the end of the line. (50 reps) |
| Walk on mats with their eyes fixed on a poster that will be away, the patient should read the sentences written on the poster. (30 reps) |
| **13ª Step** |
| Patient walks on a line drawn on the ground and in the middle of the route to and performs a 360 ° and continue the route until the end of the line, always look in the same setting. (30 reps) |
| Patient foot and then sitting in a circle, each with a chair. The therapist will be at the center of the circle with a tambourine or other sound instrument in the hands. When the therapist playing the tambourine above her head, patients should get up. When playing the tambourine under the head the patient should sit, always with his gaze fixed on the tambourine. (30 reps) |
| Patients should form rows (columns) with the same number of patients, legs apart (open) and a ball in hand, the first of each row, passing the ball from hand to hand, under the legs. When the ball reaches the last in line, everybody turn to the other side and start again. (30 reps) |
|  |
| **14ª Step** |
| Patient sitting rises focusing an object that will be on hand therapist in front of what drives the object, and the patient will have to perform sets of squats. (30 reps) |
| Patient performs standing hip abduction, keeping only supported on a limb. Focusing on a fixed point that is in front of you. (30 reps) |
| Patient standing with eyes closed, with a foot on the ball and the other serving you support on a mattress, and can not let the ball escape from his control. |
| **15ª Step** |
| Patient walk straight hitting the ball on the floor and fixing his gaze on an object at the end of the line and the end of the back straight all the way. (50 reps) |
| Patient with bended knees in sandy terrain, perform circumduction shoulder with closed eyes. (50 reps) |
| Patient walking on a straight line on uneven ground at the end of the line will have four cones, where the patient must make the diversion of four and return the previous line. (50 reps) |
| Walking on mats, staring at a poster that will be away, the patient should read the sentences written on the poster. (30 repetitions). |
